# Prevalence and characteristics of primary left-sided valve disease in a cohort of 15,000 patients undergoing echocardiography studies in a tertiary hospital in Uganda

**DOI:** 10.1186/s12872-018-0813-5

**Published:** 2018-05-04

**Authors:** Joselyn Rwebembera, William Manyilirah, Zhang Wan Zhu, Juliet Nabbaale, Judith Namuyonga, Isaac Ssinabulya, Sulaiman Lubega, Peter Lwabi, John Omagino, Emmy Okello

**Affiliations:** 1 Uganda Heart Institute, Kampala, Uganda; 2 School of Medicine Makerere University, Kampala, Uganda

**Keywords:** Primary, Left-sided, Valve disease, Rheumatic, Non-rheumatic, Uganda

## Abstract

**Background:**

Although rheumatic heart disease remains the leading cause of valve heart disease (VHD) in developing countries, other forms of valve disease have been over shadowed and not regarded as a public health problem. However, several facts suggest that the role of non-rheumatic VHD as a significant cardiovascular disease should be reconsidered. We aimed to assess the prevalence and characteristics of different forms of primary left sided valve diseases from a series of 15,009 echocardiographic studies.

**Methods:**

This was a retrospective review of echocardiographic reports for studies performed between January 2012 and December 2013 (24 months) at Uganda Heart Institute. All patients with primary left-sided valve disease were classified into one of five major diagnostic categories and in each diagnostic category; patients were sub-classified into stages A-D of primary valve disease as defined by the American College of Cardiology.

**Results:**

Three thousand five hundred eighty-two echocardiography reports qualified for final data analysis. The “sclerotic valve changes with normal valve function”, a Stage A sub-class of “degenerative valve disease” overwhelmingly overshadowed all the other diagnostic categories in this stage. “Rheumatic Heart Disease”, “Degenerative Valve Disease”, “Bicuspid Aortic Valve”, “Mitral Valve Prolapse” and “Endomyocardial Fibrosis” diagnostic categories accounted for 53.0%, 41.8%, 2.2%, 1.4% and 1.7% respectively in stages B-D of primary VHD. Rheumatic heart disease disproportionately affected the young, productive age groups. It was the major risk factor for infective endocarditis; and was the indication for valve surgery in 44 of 50 patients who had undergone valve replacement procedures.

**Conclusions:**

We acknowledge that rheumatic heart disease remains a leading cause of progressive and severe primary left-sided valve disease among young adults in Uganda. But we bring to light the contemporary footprints of other forms of primary valve disease that require coordinated multidisciplinary approach to research, education and clinical management to ensure improved patient outcomes.

## Background

Valvular heart disease is classically predominated by rheumatic heart disease (RHD) in the young in low-resource settings and by calcific aortic valvluar disease in the elderly in high-resource settings [1–3]. In the last decade, studies from across the African continent have begun to provide a better understanding of RHD across the continent, with important advances in epidemiology, diagnosis, and outcomes [4–11].

However, the last two decades have witnessed a substantial increase in life expectancy in sub-Saharan Africa [12], and more of the population is living past the 7th decade of life. It stands to reason that the etiology of valvular heart disease may also be shifting, as has been documented in middle-income countries [13–15].

The Uganda Heart Institute (UHI) provides cardiology tertiary referral for the country of Uganda, with diagnostic echocardiography and both pediatric and adult cardiologists with expertise in echocardiographic performance and interpretation. The objective of this study was to classify the burden of primary left-sided valvular heart disease across the lifespan in Uganda, through retrospective study of a large cohort of patients evaluated at UHI over a two-year period.

## Methods

### Study design

This was a retrospective review of echocardiographic reports for studies performed between January 2012 and December 2013 (24 months) at UHI.

### Study site

UHI is a specialized tertiary centre for cardiovascular care in Uganda, located within Mulago Hospital Complex, the country’s national referral hospital and teaching hospital for Makerere University Medical School. The adult (≥ 13 years) echocardiography laboratory serves approximately 10,000 patients per year with three fully functional echocardiography machines (2 x GE medical Systems Vivid 7 Dimension - GE Vingmed Ultrasound AS N-3190 Horten, Norway and 1 x Phillips IE33 – Phillips Ultrasound Bothell WA, 98041 USA).

### Study population

**Fig. 1:**
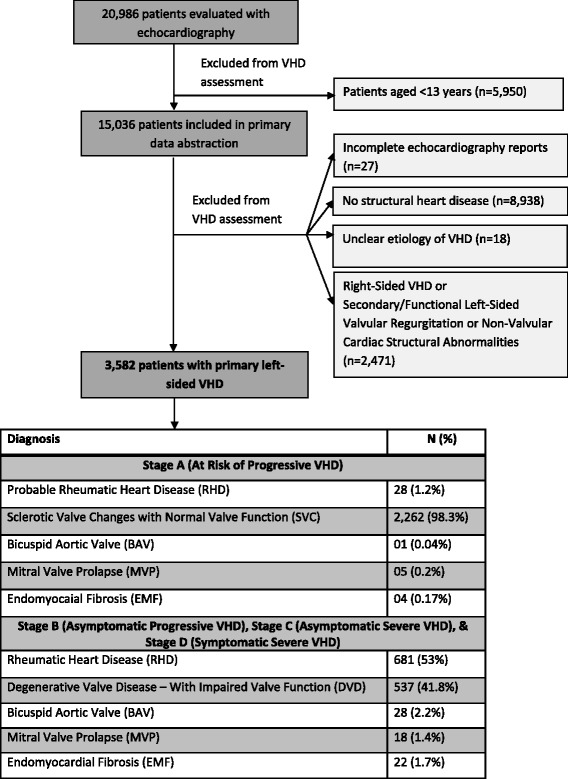
Flow chart of the echocardiography study selection process. Legend: Echocardiography report selection was based on age of the patients at the time of the echocardiography study and completeness of the echocardiography reports

The study population consisted of adults who had an echocardiogram performed at UHI during the study period. Only the first echocardiographic report from each patient during the study period was utilized for data collection. Retrospective echo report review was performed for all patients ≥13 years. Patients with an incomplete echocardiography report, those found to have no structural heart disease, those with unclear etiology of valvular disease, those with secondary/functional left-sided valvular regurgitation and those with non-valvular cardiac structural abnormalities were excluded from further data analysis (Fig. 1).

### Echocardiography procedures

Echocardiograms were performed by adult cardiologists or cardiology fellows in accordance with the American Society of Echocardiography guidelines [16, 17]. Although these echocardiographic studies were performed in 2012 and 2013, we reviewed the valve parameters provided in the echocardiography reports and graded the severities of valve dysfunction (regurgitation and/or stenosis) according to the 2014 and 2017 guidelines for the management of patients with valvular heart disease [18–21] (Table 1). The etiology of the valve dysfunction remained the same as that which was reported in the original reports. Care was always taken during the echocardiography studies to exclude secondary mitral regurgitation due to dilated cardiomyopathy and ischemic heart disease before making a diagnosis of “primary mitral regurgitation” [18, 21].

**Table 1:** Grading of mitral and aortic valve regurgitant and stenotic lesions

Valve lesion	Grading method	Mild	Moderate	Severe
Mitral stenosis	Valve area	≥2.0 cm^2^	1.6–1.9 cm^2^	≤1.5 cm^2^
Primary mitral regurgitation	Vena contracta width	< 0.3 cm	0.3–0.69 cm	≥0.7 cm
	Regurgitant fraction	< 30%	30–49%	≥50%
	Regurgitant volume	< 30 mls	30-59 mls	≥60 mls
	Effective regurgitant orifice area	< 0.2 cm^2^	0.2–0.39 cm^2^	≥0.4 cm^2^
Aortic sclerosis	Peak velocity	< 2.5 m/s		
Aortic stenosis	Peak velocity	2.6–2.9 m/s	3.0–4.0 m/s	≥4.0 m/s
	Mean gradient	< 20 mmHg	20-39 mmHg	≥40 mmHg
	Valve area by continuity equation	> 1.5cm^2^ or indexed AVA > 0.85 cm^2^/m^2^	1.0–1.5cm^2^ or indexed AVA 0.6–0.85 cm^2^/m^2^	≤ 1.0cm^2^ or indexed AVA ≤ 0.6 cm^2^/m^2^
Aortic regurgitation	Vena contracta width	< 0.3 cm	0.3–0.6 cm	≥0.6 cm
	Central jet width/LVOT width	< 25%	25–64%	≥65%
	Regurgitant fraction	< 30%	30–49%	≥50%
	Regurgitant volume	<30 mls	30–59 mls	≥60 mls
	Effective regurgitant orifice area	< 0.1 cm^2^	0.1–0.29 cm^2^	≥0.3 cm^2^

### Diagnostic classification

Patients with primary left-sided valvluar heart disease were categorized into one of 5 major categories:

1. Rheumatic Heart Disease (RHD), which was diagnosed according to the 2012 World Heart Federation criteria [22].
2. Degenerative valve disease (DVD), which represented: a) Sclerotic valve changes but with normal valve function: This required presence of mitral and/or aortic leaflet sclerosis - seen as calcium deposits on the leaflets without involvement of the commissures and without valve incompetence or stenosis [16, 20, 23, 24]; or b) Degenerative valve changes with impaired valve function, which required sclerosis/calcification of the mitral and/or aortic valvular apparatus with resultant mild to severe valve incompetence or stenosis and in the absence of rheumatic features [16–21, 23, 24].
3. Bicuspid Aortic Valve (BAV), which was defined by the finding of fusion of any 2 of the aortic valve cusps with or without a raphe [25, 26]. All aortic valves that met this anatomical definition were included in this diagnostic category regardless of the functional status.
4. Primary mitral valve prolapse (MVP), for which the standard echocardiographic definitions of classic and non-classic MVP were utilized [17, 21, 27, 28].
5. Endomyocardial Fibrosis (EMF), whose diagnosis was based on the typical echocardiographic features that have been described [29, 30].

In each of these diagnostic categories, patients were further classified according to the 2014 ACC/AHA staging of primary VHD [18].

1. Stage A represents patients with risk factors for development of progressive VHD, and in this series included probable RHD (stage A of RHD), sclerotic valve changes but with normal valve function (stage A of DVD), bicuspid aortic valve with normal valve function, mitral valve prolapse without mitral valve incompetence and endomyocardial fibrosis without mitral/aortic valve dysfunction [18].
2. Stages B, C and D represent mild to moderate asymptomatic VHD, severe asymptomatic VHD, and severe symptomatic VHD respectively [18]. In this series, these stages included mild, moderate and severe valve incompetence and/or stenosis in each of the 5 diagnostic categories. Patients who had undergone surgical procedures for the correction of valve abnormalities were categorized in group D.

### Other echocardiographic assessments

Examination of native or prosthetic valves for echocardiographic features of past or current infective endocarditis was performed according to standard guidelines [31, 32]. Prosthetic valves were evaluated according to the 2009 American Society of Echocardiography’s Guidelines for the echocardiographic evaluation of prosthetic valves [31].

### Data management

Data was initially captured in Epi info 3.0 and later exported to Stata 12.0. Continuous variables are presented as means ± standard deviation. Categorical variables are presented as proportions.

## Results

**Fig. 2:**
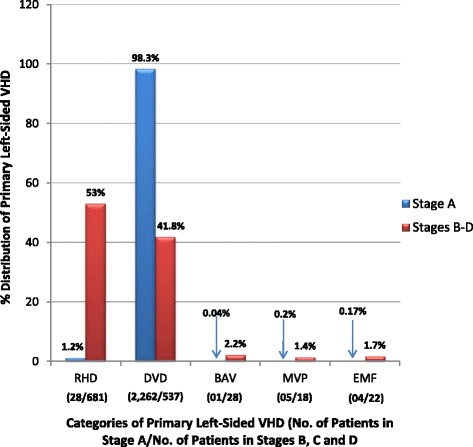
The 5 major echocardiographic diagnostic categories. Legend: Stage A: Patients with risk factors for development of progressive VHD; Stage B: Progressive VHD (asymptomatic mild to moderate VHD); Stage C: Asymptomatic severe VHD; D: Symptomatic severe VHD

Of 20,986 echocardiographic studies performed during the study period, 3582 were included in the data analysis (Fig. 1). Over 60% of the 3582 patients were females (Table 2). The diagnostic category of “sclerotic valve changes with normal valve function” accounted for 98% of the primary valve diseases in Stage A while rheumatic heart disease accounted for over half of the primary valve diseases in Stages B to D (Fig. 2).

**Table 2:** Participant demographics of the 3582 patients

Characteristics of the 3582 patients	Number of participants (%)
Age groups (years)	
13–19	251 (7.0%)
20–29	162 (4.5%)
30–39	128 (3.6%)
40–49	404 (11.3%)
50–59	503 (14.0%)
60–69	612 (17.1%)
70–79	733 (20.5%)
80–89	714 (20.0%)
90–99	70 (2.0%)
≥100	05 (0.1%)
Sex	
Male	1329 (37.1%)
Female	2253 (62.9%)

### “Stage a” primary left-sided valve diseases

“Sclerotic valve changes with normal valve function” overshadowed all the other valve diseases in this stage (Fig. 2). All of the patients with sclerotic valve changes with normal valve function were aged ≥40 years and 56% were males. There were much smaller numbers of patients “at risk” of progressive RHD, BAV, MVP and EMF-related valve dysfunction.

### “Stages b, c and d” primary left-sided valve diseases

RHD was the leading cause of valve disease in these categories and was closely followed by DVD. The other diagnostic categories occurred in much smaller numbers.

**Fig. 3:**
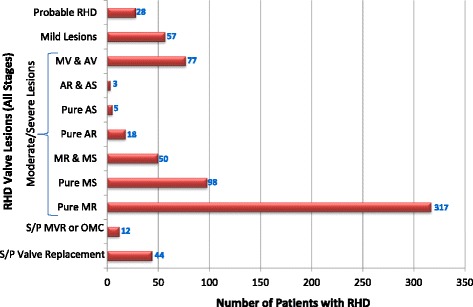
Number of patients with RHD valve lesions (all stages). Legend: Mild lesions” represented definite RHD with mild mitral and/or aortic valve incompetence and/or stenosis. In the categories of “Pure AS” and “Pure AR”, patients always had echocardiographic features of rheumatic involvement of the mitral valve (with trivial to mild mitral valve dysfunction) which supported the diagnosis of RHD. “Pure MR”: Isolated moderate to severe mitral regurgitation; “Pure MS”: Isolated moderate to severe mitral stenosis; “MR & MS”: mixed mitral valve disease – coexistent moderate to severe mitral regurgitation and mitral stenosis; “AR & AS”: mixed aortic valve disease – moderate to severe aortic regurgitation and stenosis; “MV & AV”: mixed mitral and aortic valve disease - coexistent moderate to severe mitral and aortic valve disease

**Fig. 4:**
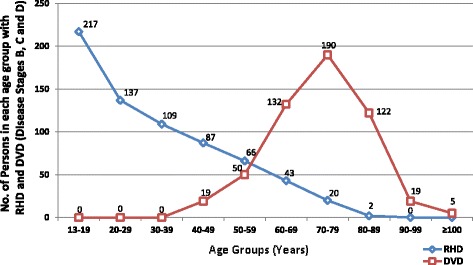
Number of persons in different age groups with RHD and number of persons in different age groups with DVD. Legend: RHD: Rheumatic Heart Disease, DVD: Degenerative Valvular Disease

**Fig. 5:**
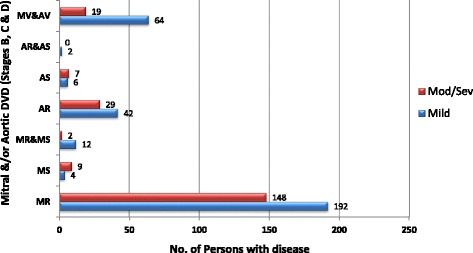
Frequency of degenerative valve lesions (Stages B-D). Legend: MR: Mitral regurgitation alone; MS: Mitral stenosis alone; MR & MS: Mixed mitral valve regurgitation and stenosis; AR: Aortic regurgitation alone; AS: Aortic stenosis alone; AR & AS: Mixed valve regurgitation and stenosis; MV & AV: Mixed mitral and aortic degenerative valve disease

**Fig. 6:**
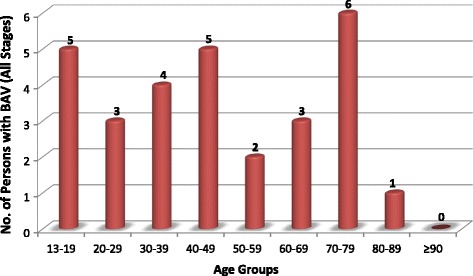
Age-group distribution of bicuspid aortic valve (all stages). Legend: Number of persons in different age groups with bicuspid aortic valve, with or without valve dysfunction

**Fig. 7:**
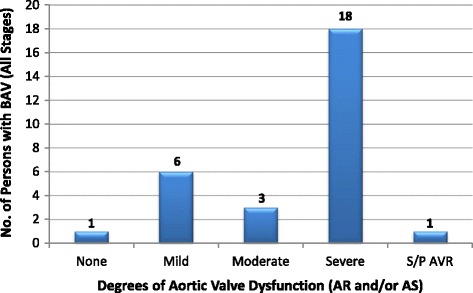
Functional status of the aortic valve among patients with bicuspid aortic valve (all stages). Legend: AS: Aortic Stenosis; AR: Aortic Regurgitation; S/P AVR: Status Post Aortic Valve Replacement

**Fig. 8:**
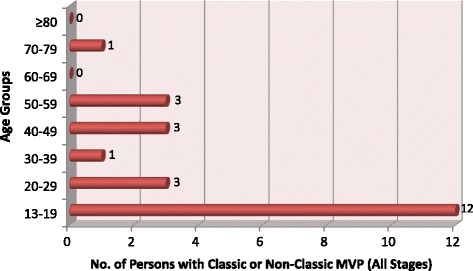
Age-group distribution of mitral valve prolapse (all stages). Legend: Persons with classic or non-classic mitral valve prolapse, with or without mitral valve regurgitation in different age groups

- Rheumatic Heart Disease
  Stages B-D of RHD included mild, moderate and severe RHD valve lesions. We also included patients with RHD who had undergone valve replacement procedures for RHD valve dysfunction and those who had undergone mitral valve repair or open mitral commissurotomy for rheumatic mitral valve disease (Fig. 3). Patients who had undergone valve replacement, mitral valve repair or open mitral commissurotomy were classified as such regardless of the functional status of left sided native or prosthetic valves at the time of echocardiography. Isolated mitral regurgitation was the most common rheumatic valve lesion. RHD disproportionately affected the young under-40 years age group, with the ‘number of cases per age group’ falling below that of DVD in the 5th decade of life (Fig. 4).
- Degenerative Valve Disease
  Calcific degenerative mild to severe valve disease was the second common diagnostic category, following closely after RHD (Fig. 2). The ‘number of cases per age group’ increased with age, peaking in the 70–79 year age group (Fig. 4). Mitral regurgitation was the most predominant degenerative valve lesion (Fig. 5).
- Bicuspid Aortic Valve
  Twenty-nine patients were reported to have bicuspid aortic valves. Sixteen (55%) were males, and there was a fair distribution among all the age groups (Fig. 6). Majority of the affected patients had severe aortic valve dysfunction while 1 patient had undergone aortic valve replacement (Fig. 7). Two patients had co-existing coarctation of the aorta, 2 patients had ascending aortic aneurysms – a manifestation of bicuspid aortopathy while 1 patient had echocardiographic evidence of aortic valve endocarditis. There were no reports of other congenital structural valve abnormalities like cleft mitral valve, parachute mitral valve or sub-mitral aneurysms over the 24 months.
- Primary Mitral Valve Prolapse
  Twenty three patients had definite primary classic or non-classic mitral valve prolapse. Majority of these were teenagers (Fig. 8). All the cases were reported to be myxomatous in origin. Three patients were reported to have co-existent aortic leaflet prolapse but with preserved aortic valve function in all the cases. Of the 23 individuals with MVP, 3 had severe, 8 had moderate, 7 had mild while 5 had no mitral valve regurgitation.
- Endomyocardial Fibrosis
  Twenty-two patients had moderate to severe mitral valve regurgitation due to involvement of the mitral valve in left ventricular or biventricular endomyocardial fibrosis. EMF was exclusively diagnosed in the 13–39 years age groups, and 72% of the patients were females.

### Other findings

**Fig. 9:**
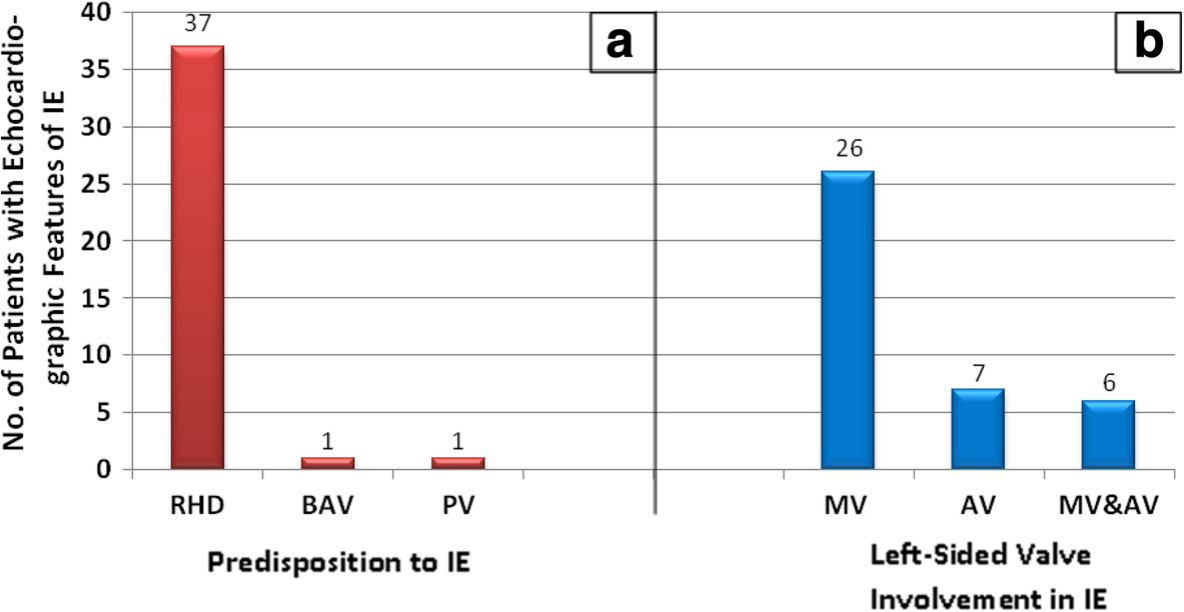
Risk factors for and patterns of valve involvement in infective endocarditis. Legend: **a**: Cardiac structural abnormalities that likely predisposed patients to infective endocarditis. RHD: Rheumatic Heart Disease, BAV: Bicuspid Aortic Valve, PV: Prosthetic Valves; **b**: Left sided valve involvement in infective endocarditis. MV: Only mitral valve involvement; AV: Only aortic valve involvement; MV & AV: Both mitral and aortic valves involved in infective endocarditis

**Fig. 10:**
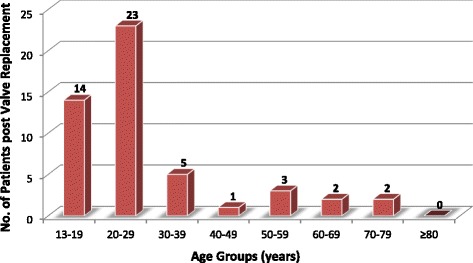
Number of persons with prosthetic valves in different age groups. Legend: Persons with prosthetic valves (mechanical or bioprosthetic)

- Infective Endocarditis
  Infective endocarditis is not presented as a separate diagnostic category because there were no reports of “primary” endocarditis. All the cases of left-sided infective endocarditis that were reported were super-imposed on another form of primary valve disease. Echocardiographic evidence of past or active mitral and/or aortic valve infective endocarditis was reported in 39 patients. RHD was the predominant predisposing factor and the mitral valve was most commonly affected (Fig. 9). One patient had an aortic root abscess while the rest of the patients had mitral and/or aortic valve vegetations. A description of further work up to confirm active infection and the causative organisms is beyond the scope of this paper.
- Prosthetic Valves
  Fifty patients had undergone mitral and/or aortic valve replacement procedures. RHD was overwhelmingly the leading indication for valve replacement and mechanical prosthetic valves were mostly utilized (Table 3). The majority of valve replacements were performed when patients were under 30 years of age (Fig. 10). All the prosthetic valves were functioning normally. One patient had evidence of vegetations on a mechanical mitral prosthesis but the valve function was normal.

**Table 3:** Indications for valve replacement, valve(s) replaced, and types of valves used for replacement

Indications for valve replacement	
Indication	No. of patients
Rheumatic heart disease	44
Aortic root disease with aortic regurgitation	2
Dilated cardiomyopathy with functional mitral regurgitation	1
Bicuspid aortic valve	1
Calcific aortic stenosis	2
Valve(s) replaced	
Valve(s)	No. of patients
Mitral valve only	26
Aortic valve only	10
Mitral & Aortic valves	13
Mitral, aortic and tricuspid valves	1
Types of prosthetic valves	
Type of prosthetic valve	No. of patients
Mechanical valves	44
Bioprosthetic valves	5
Autograft (ross procedure)	1

## Discussion

In this study, we describe the burden of a variety of forms of primary left-sided valve disease from a large series of echocardiographic studies in a tertiary cardiac care centre in Uganda.

We demonstrated a large number of patients in this series with “sclerotic valve changes with normal valve function”. This early stage of leaflet changes is recognized as “Stage A” or the “initiation stage” of progressive DVD, and indicates a population that is at risk of developing progressive valve stenosis or regurgitation [18–21, 24]. Progressive valve disease develops in approximately 10–15% of patients with valve sclerotic changes, and this category of patients has been shown to have an increased risk of cardiovascular events over 5 years [24]. Although previous randomized clinical trials of lipid-lowering therapy in adults with mild-to-moderate aortic stenosis showed no significant effect on disease progression or aortic-valve events [33], this early “initiation” stage remains a potential opportunity for medical intervention to prevent or delay disease progression.

Rheumatic heart disease was the leading cause of primary left-sided valve disease with valve dysfunction. There is already a bulk of descriptive data on patterns, presentations, and complications of RHD in Africa and – in particular, from the current study site by the same working group [4–11].

In high income countries, VHD is typically degenerative and is regarded as an emerging public health problem [2]. Degenerative valve disease closely tallied behind RHD among the diagnostic categories with valve dysfunction in this echocardiographic series. In what appears to be quite a similar black South-African study population, Sliwa et al. [14] reported a prevalence of 21% of DVD among structural valve abnormalities. Differences in reporting methods and slight variations in the study populations do not permit direct comparison, but it is evident that the problem of DVD is not as minor in developing countries as it is assumed to be. With improvement in socioeconomic living conditions and increasing life expectancy in Africa [12]; more cases of DVD should be expected as we experience our share of the epidemiological transition of VHD.

Bicuspid aortic valve (BAV) is the commonest congenital cardiac abnormality in the general population with an estimated prevalence of 1–2% [34]. Although the findings in this study are far from being representative of the general population of Uganda, we bring into the limelight the existence of a pathology in a setting where there is barely any prior literature recording. In Uganda, only a singleton remote documentation by Somers and Rankin in 1962 [35] of a case of aortic coarctation co-existing with BAV can be found. The medical fraternity needs to master its natural progression, as evidenced by the proportions of patients with mild to severe degrees of valve dysfunction, and other clinical components of this pathology such as indications and timing of surgical intervention, infective endocarditis, and bicuspid aortopathy, as demonstrated in this echocardiographic series.

Primary MVP contributed 1.4% to the pool of primary valve diseases in stages B-D. Despite the study population and reporting differences, our findings rhyme closely with those of Sliwa et al. [14] who reported a prevalence of MVP of 1% among 481 black patients with structural heart disease. Majority of the patients with MVP were young and had mild to moderate mitral regurgitation, similar to what several population-based studies have demonstrated. Severe mitral regurgitation was less common [27, 36].

Recognized as the country where EMF was first described [37, 38], Uganda has remained an endemic region for the disease. EMF is one of the neglected cardiovascular diseases whose etiology and pathogenesis remain elusive, the natural history is incompletely understood and therefore, no effective therapy is currently available [29, 39]. It remains an important cause of heart failure in endemic areas, comparable to rheumatic heart disease. The mechanisms by which heart failure develops are multifactorial with valvular involvement and dysfunction inclusive. Concerted efforts from local scientists, communities, governments and international collaborations are needed for renewed interest in this neglected tropical disease.

Infective endocarditis is an important component of VHD, with the predisposing factors in developing countries strikingly different from those in developed countries. Despite its clinical impact and poor outcomes in our setting, data on this subject are missing or sparse in many low- and middle-income regions [40]. Previous studies in sub-Saharan Africa including the current study site have reported on the burden of infective endocarditis (IE) among patients with RHD [11, 14, 41, 42]. A few prospective and retrospective descriptive studies on etiology and risk factors for IE have been performed in some parts of Africa, and they report RHD to be the leading risk factor for IE [43, 44]. In keeping with these previous reports, RHD was the identifiable predisposition in 94% of patients with echocardiographic features of IE in our study. The only detailed description of IE in Uganda dates back to 1972 [45], at which time a preponderance of RHD as the predisposing factor was found in 65% of patients in the clinically diagnosed series. For purposes of achieving up-to-date local clarity, our team is currently conducting a prospective study of the risk factors and etiology of infective endocarditis in Mulago National Referral Hospital.

RHD was the leading indication for valve surgery in this series as opposed to developed countries where degenerative valve disease takes the lead. Valve surgery is becoming more accessible in Uganda and therefore the number of patients with prosthetic valves is consistently on the rise. By definition, valve disease is still present after interventional therapy and prosthetic valves are a recognized independent entity of valve heart disease. The clinical issues surrounding the timing of valve surgery, prosthetic valve selection, prosthetic valves and pregnancy, anticoagulation and infective endocarditis pose a new frontier in a developing country that all practicing clinicians must get conversant with.

### Strengths and limitations

This descriptive study provides vital information that should act as a basis for more focused and analytical studies in the field of valve heart disease in Uganda and developing countries at large. The main limitation of this study is its retrospective nature and thus the inherent shortcoming of incomplete data among others. Also, these findings do not reflect the burden of valve heart disease in the general population of Uganda.

## Conclusions

Over the past years, a picture has been painted which gives a reflection that “valve heart disease in Africa is synonymous with rheumatic heart disease in Africa”. Our bringing to the limelight by way of publication of the contemporary footprints of other forms of VHD in significant proportions suggests that this assumption is not true. National and continental programs already exist for rheumatic heart disease. A similar coordinated multidisciplinary approach to research, education and clinical management is now needed to ensure improved outcomes for patients with other forms of VHD.

## Abbreviations

AR: Aortic regurgitation
AS: Aortic stenosis
BAV: Bicuspid aortic valve
DVD: Degenerative valve disease
EMF: Endomyocardial fibrosis
IE: Infective endocarditis
MR: Mitral regurgitation
MS: Mitral stenosis
MVP: Mitral valve prolapse
RHD: Rheumatic heart disease
UHI: Uganda Heart Institute
VHD: Valve heart disease
